# Super-resolution reconstruction, recognition, and evaluation of laser confocal images of hyperaccumulator *Solanum nigrum* endocytosis vesicles based on deep learning: Comparative study of SRGAN and SRResNet

**DOI:** 10.3389/fpls.2023.1146485

**Published:** 2023-03-21

**Authors:** Wenhao Li, Ding He, Yongqiang Liu, Fenghe Wang, Fengliang Huang

**Affiliations:** ^1^ School of Electrical and Automation Engineering, Nanjing Normal University, Nanjing, China; ^2^ Jiangsu Provincial Key Laboratory of Materials Cycling and Pollution Control, School of Environment, Nanjing Normal University, Nanjing, China

**Keywords:** laser confocal, deep learning, plant cell, endocytic vesicle, super-resolution reconstruction, SRGAN, SRResNet

## Abstract

It is difficult for laser scanning confocal microscopy to obtain high- or ultra-high-resolution laser confocal images directly, which affects the deep mining and use of the embedded information in laser confocal images and forms a technical bottleneck in the in-depth exploration of the microscopic physiological and biochemical processes of plants. The super-resolution reconstruction model (SRGAN), which is based on a generative adversarial network and super-resolution reconstruction model (SRResNet), which is based on a residual network, was used to obtain single and secondary super-resolution reconstruction images of laser confocal images of the root cells of the hyperaccumulator *Solanum nigrum*. Using the peak signal-to-noise ratio (PSNR), structural similarity (SSIM) and mean opinion score (MOS), the models were evaluated by the image effects after reconstruction and were applied to the recognition of endocytic vesicles in *Solanum* nigrum root cells. The results showed that the single reconstruction and the secondary reconstruction of SRGAN and SRResNet improved the resolution of laser confocal images. PSNR, SSIM, and MOS were clearly improved, with a maximum PSNR of 47.690. The maximum increment of PSNR and SSIM of the secondary reconstruction images reached 21.7% and 2.8%, respectively, and the objective evaluation of the image quality was good. However, overall MOS was less than that of the single reconstruction, the perceptual quality was weakened, and the time cost was more than 130 times greater. The reconstruction effect of SRResNet was better than that of SRGAN. When SRGAN and SRResNet were used for the recognition of endocytic vesicles in *Solanum nigrum* root cells, the clarity of the reconstructed images was obviously improved, the boundary of the endocytic vesicles was clearer, and the number of identified endocytic vesicles increased from 6 to 9 and 10, respectively, and the mean fluorescence intensity was enhanced by 14.4% and 7.8%, respectively. Relevant research and achievements are of great significance for promoting the application of deep learning methods and image super-resolution reconstruction technology in laser confocal image studies.

## 1 Introduction

Confocal laser scanning microscopy (CLSM) has been used in cellular molecular biology (Stephens and Allan, 2003), 3D imaging of materials (Ji et al., 2021), material porosity determination (Mauko et al., 2009), and reaction process visualization (Onuma et al., 2017). It has also been used to identify the distribution of fillers in a polymer matrix (Zhong et al., 2017), including the examination of the structure of tobacco microsporocytes involved in the intercellular migration of nuclei (cytomixis), as well as to study live cell chiral molecular interactions (Mursalimov et al., 2017; Stachelek et al., 2022). For the purpose of cellular molecular biology, the high sensitivity, high spatial resolution, and super-optical sectioning ability of CLSM make it an ideal tool for studying biological systems, including membranes, tissues, and cell structures. It can be used to carry out quantitative fluorescence determination in tissues and cells, physicochemical determinations in cells, and long-term observation of cell migration and growth. It can also provide visual evidence for the study of plant growth processes and phytoremediation processes and mechanisms (Stephens and Allan, 2003; Nwaneshiudu et al., 2012; Paddock and Eliceiri, 2014; Hall et al., 2016; Khan et al., 2020; He et al., 2021; Zhang et al., 2022). One important application is the filming of plant cell endocytosis. Endocytosis plays an important role in information transfer, nutrient uptake, and pollution remediation in plants. The visualization of endocytosis in plant cells can provide important theoretical support for the elucidation of life processes in plants (Fan et al., 2015; He et al., 2023). The number of endocytosis vesicles is important information, and currently, endocytosis cell counting is mainly identified by manual visual counting with low levels of efficiency and large error. At present, high-resolution and clear images in laser scanning confocal microscopy are mostly obtained by adjusting the relevant parameters using acquisition software. Due to the diffraction limit in optical microscopy and the principle of point-by-point scanning in laser scanning confocal microscopy, and point-to-line and line-to-plane imaging, it is difficult to directly obtain higher or ultra-high-resolution laser confocal images using laser scanning confocal microscopes, which greatly hinders the deep mining and use of the embedded information in the images.

Super-resolution image reconstruction (SRIR) is a technology for obtaining high-resolution images from low-resolution images using algorithms. SRIR has great importance and value in practical applications such as medical imaging, satellite remote sensing, monitoring, and scientific research (Zhang et al., 2010; Isaac and Kulkarni, 2015; Courtrai et al., 2020). Traditional super-resolution reconstruction algorithms rely on basic digital image processing techniques for reconstruction, including interpolation-based, degradation-based, and learning-based super-resolution reconstruction (Shezaf et al., 2000; Yang et al., 2008; Yang et al., 2022). The rapidly developing technology of artificial intelligence has injected energy into scientific research and now plays an important role in life sciences, mathematics, chemistry, space science, and other disciplines (Yu, 2022). Deep learning, as a core technology in artificial intelligence, forms a new research field in machine learning. It simulates the human brain in analyzing and interpreting data to allow a computer to learn relevant features and make relevant predictions according to the learned features. It has obvious advantages over machine learning, which requires the manual extraction of relevant features (Yann et al., 2015). The application of deep learning to image super-resolution reconstruction is an emerging trend. In 2014, Dong et al. used a convolution neural network for single-image super-resolution reconstruction and proposed a super-resolution convolution neural network, which had a lightweight structure, could be used online, and was the best image reconstruction model at that time (Dong et al., 2016). Owing to the remarkable effects deep learning produced when applied to super-resolution reconstruction, a variety of neural networks in deep learning, such as deep convolutional neural networks (Kim et al., 2016) and generative adversarial networks (GAN) (Ledig et al., 2017; Yuan et al., 2022), have been applied to image super-resolution reconstruction.

The potential to obtain high-definition images through image super-resolution reconstruction technology has attracted the attention of many researchers (Lin et al., 2019; Yang et al., 2020; Dreier et al., 2021; Wei et al., 2022), but no reconstruction or application of laser confocal images has been reported in the literature. GAN and residual networks (ResNet) are two representative deep-learning networks. Applying the GAN-based Super-resolution Reconstruction Model (SRGAN) and ResNet-based Super-resolution Reconstruction Model (SRResNet) to laser confocal image reconstruction has strong overall importance for exploring the application of deep learning methods in laser confocal image research. In this study, deep learning was applied to laser scanning confocal microscopic images of the root cells of the hyperaccumulator *Solanum nigrum.* We compared super-resolution image reconstruction using SRGAN and SRResNet, evaluating different image quality indicators. Our results promote the intersection of artificial intelligence and botany, environmental science, ecology, and other disciplines and provide a new concept for the in-depth study of phytoremediation-related cytological characteristics.

## 2 Research methods and steps

### 2.1 Deep learning model

#### 2.1.1 SRGAN

**Figure 1:**
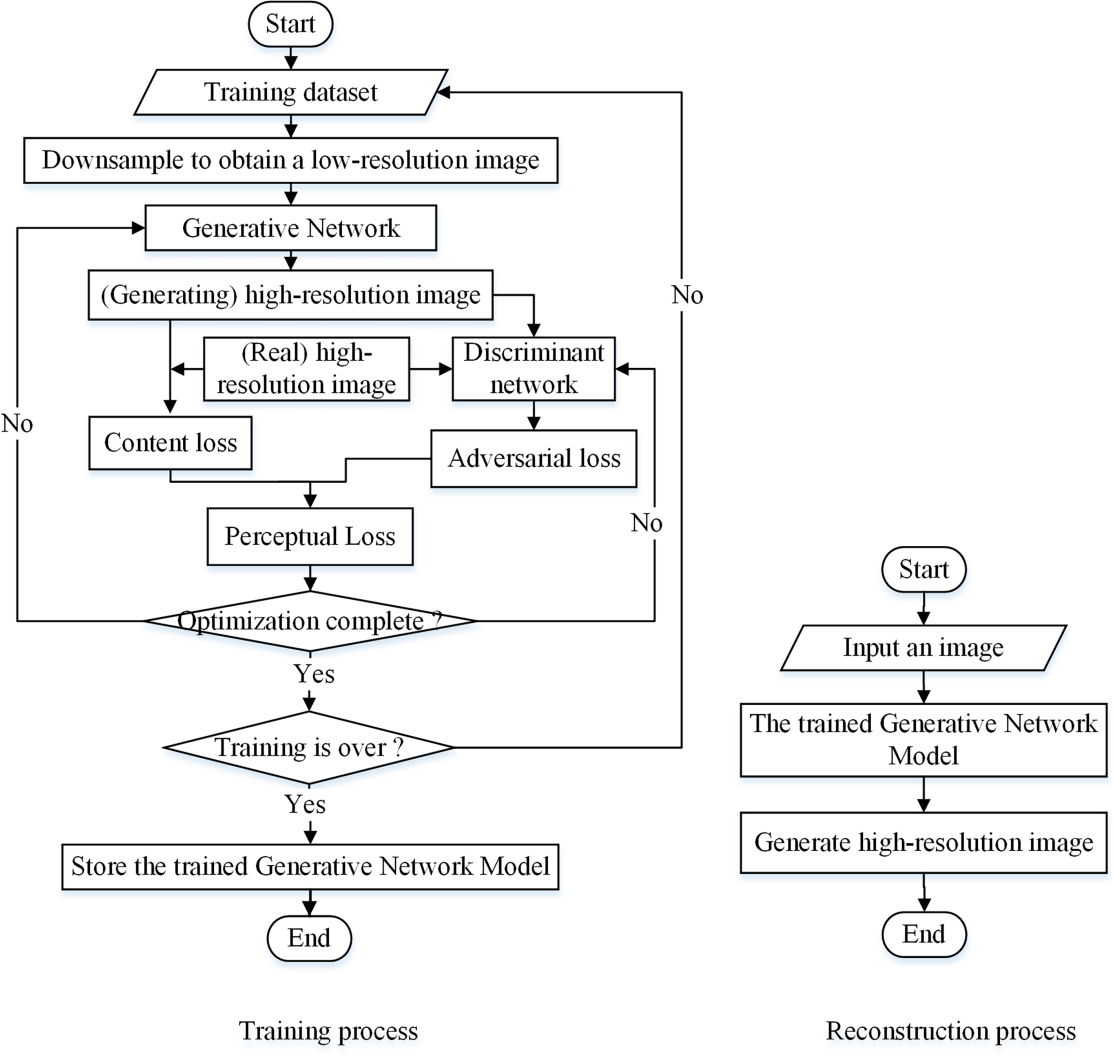
Image super-resolution reconstruction process for SRGAN.

SRGAN has the same overall structure as GAN. It is composed of a generative network and a discriminant network, and the perceived loss is used as the loss function (Ledig et al., 2017; Li and Zhang, 2022; Singla et al., 2022). The generative network of the SRGAN can take an image as an n-dimensional vector input to generate the reconstructed image output. The discriminant network determines the authenticity of the image generated by the network using a loss function. For the loss function, the perceptual loss includes content- and adversarial- loss, where the former is the mean square error (MSE) between the reconstructed high-resolution image feature map and the original image feature map, and the latter is the loss when the reconstructed high-resolution image is correctly judged by the discriminator. Perceptual loss is continuously optimized using algorithms, and the learning of the generative and discriminant models is continuously supervised. When the number of iteration rounds reaches a set value to end the training, a generative network is obtained for image reconstruction. **Figure 1** shows the process of super-resolution reconstruction of an image based on SRGAN.

#### 2.1.2 SRResNet

**Figure 2:**
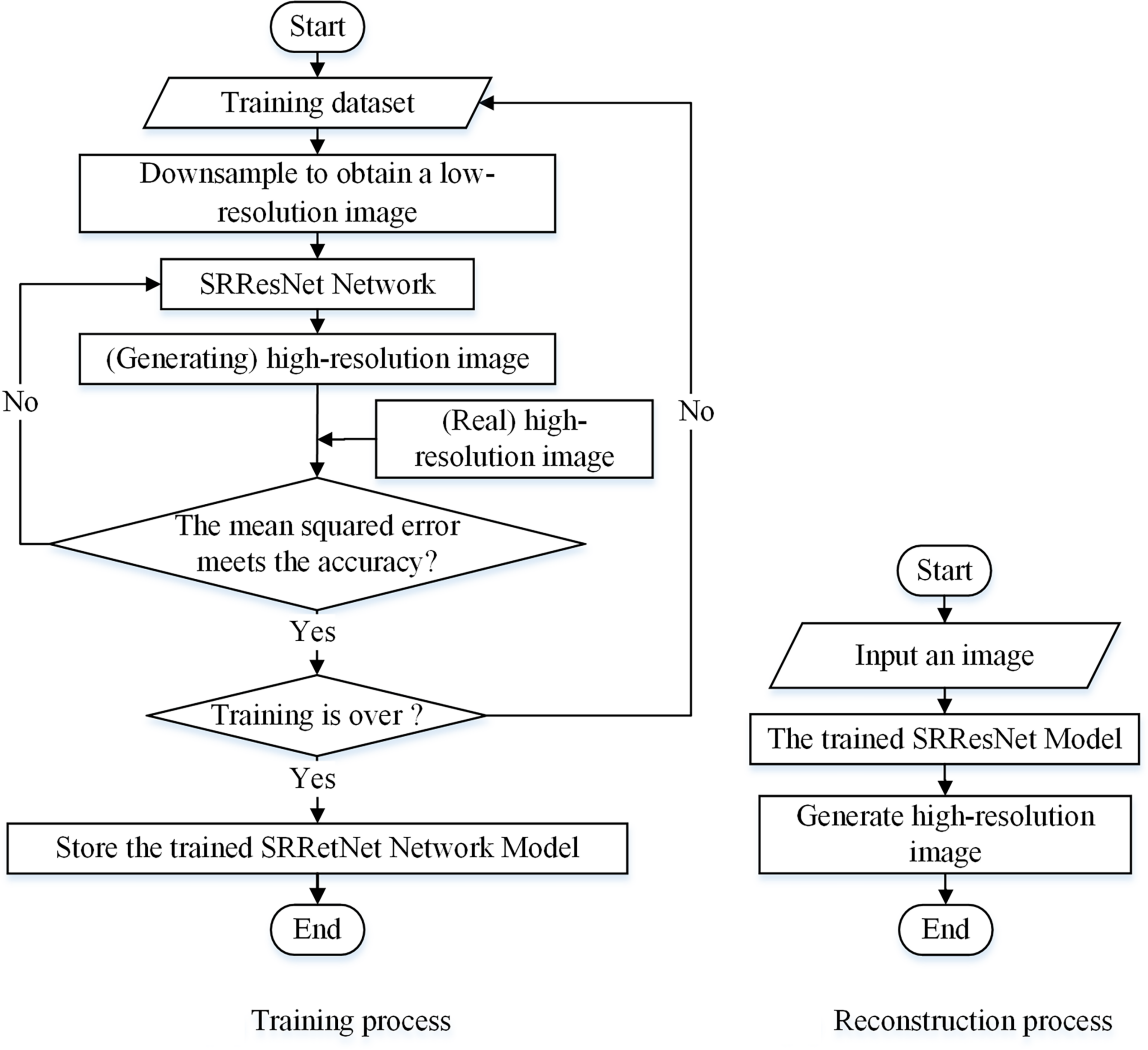
Image super-resolution reconstruction process for SRResNet.

SRResNet is based on a convolutional neural network added to a residual learning network structure (Ledig et al., 2017; Lu et al., 2022). The main body includes two parts: a deep residual network and a sub-pixel convolution network. It uses the MSE as the loss function. The deep residual network adds a residual learning module to the convolutional neural network, which effectively solves problems of accuracy degradation and gradient dispersion in the deep network, greatly deepens the number of network layers, and ensures precision. Thus, the depth and precision of the training is effectively improved, which aids efficient feature extraction and reduces image noise. The main function of the sub-pixel convolution model is to increase the size and the accuracy of the enlarged image through sample learning. SRResNet uses a low-resolution image as its input and outputs a reconstructed high-resolution image. **Figure 2** shows the process of image super-resolution reconstruction based on SRResNet.

### 2.2 Image quality evaluation indicators

In the laser confocal images reconstructed by SRGAN and SRResNet, the image quality was evaluated using three indices: peak signal-to-noise ratio (PSNR), structural similarity (SSIM), and mean opinion score (MOS). The former two indicators are objective evaluations, based on experimental numerical calculations, and the latter is a subjective evaluation based on the system of visual perception of the human eye. Taking into account the efficiency factor of practical use, the time cost of the super-resolution reconstruction of laser confocal images using different models was also considered in this evaluation.

#### 2.2.1 PSNR

PSNR represents the ratio of the maximum possible power of the signal to the destructive noise that affects its representation accuracy and is defined by the maximum pixel value and the *MSE* of the image (Singla et al., 2022).


$$\text{PSNR}=10lg\left(\frac{MAX_{I}^{2}}{MSE}\right)$$

where $MAX_{I}$ represents the maximum value of the image point color, and the maximum pixel value of the image is determined by the number of binary bits. The greater the PSNR value, the better the reconstructed image quality. For PSNR values higher than 40 dB, the image quality is excellent, while PSNRs of 30–40 dB indicate that image quality is good.

#### 2.2.2 SSIM

SSIM is a quality-evaluation framework based on structural information, which comprehensively considers the brightness, contrast, and structural information of an image (Wang et al., 2004). It uses the image mean value as brightness estimation, standard deviation as contrast estimation, and covariance as the structural similarity extent estimation. The range of SSIM is 0–1, where higher values, indicate better reconstructed image qualities.


$$\text{SSIM}\left(x,y\right)={\left[l\left(x,y\right)\right]}^{\alpha }{\left[c\left(x,y\right)\right]}^{\beta }{\left[s\left(x,y\right)\right]}^{\gamma }$$

$$l\left(x,y\right)=\frac{2\mu_{x}\mu_{y}+c_{1}}{\mu_{x}^{2}+\mu_{y}^{2}+c_{1}}$$

$$c\left(x,y\right)=\frac{2\sigma_{xy}+c_{2}}{\sigma_{x}^{2}+\sigma_{y}^{2}+c_{2}}$$

$$s\left(x,y\right)=\frac{\sigma_{xy}\mu_{y}+c_{3}}{\sigma_{x}\sigma_{y}+c_{3}}$$

where $l\left(x,y\right)=\frac{2\mu_{x}\mu_{y}+c_{1}}{\mu_{x}^{2}+\mu_{y}^{2}+c_{1}}$ represents the brightness comparison of images, $c\left(x,y\right)=\frac{2\sigma_{xy}+c_{2}}{\sigma_{x}^{2}+\sigma_{y}^{2}+c_{2}}$ represents the contrast comparison of the images, $s\left(x,y\right)=\frac{\sigma_{xy}\mu_{y}+c_{3}}{\sigma_{x}\sigma_{y}+c_{3}}$ represents the structural comparison of images, *μ* represents the mean value, *σ* represents the standard deviation, $\sigma_{xy}$ represents the covariance, and $c_{i}\left(i=1,2,3\right)$ is a constant.

#### 2.2.3 MOS

MOS measures image quality as scored by professionals in related fields who observe images with their eyes (Singla et al., 2022). It is a comprehensive evaluation of images by observers in terms of color, clarity, noise, texture, and so on. Originally used as a criterion for evaluating the quality of a compressed voice, it came to be used to evaluate image quality. The range of MOS is 1–5 points; the larger the score, the better the image quality and the higher the clarity. Usually, several professionals are selected to score image quality, and the average score is taken as the final scoring result.

### 2.3 Research Steps

#### 2.3.1 Sample preparation and observation of endocytosis

The test samples were collected from *Solanum nigrum* grown hydroponically for 4 weeks in a greenhouse (photoperiod: 16-h/8-h day/night, light intensity: 100 μmol/m^2^/s, temperature: 22°C) in 1/2 Hoagland nutrient solution. About 1 cm fresh *Solanum nigrum* root was cut, and than in 0.5 mL of 10 μM FM4-64 dye (4-{6-[4-(Diethylamino)phenyl]-1,3,5-hexatrien-1-yl}-1-[3-(triethylammonio)propyl]pyridinium dibromide) was stained for 30 min. Then the samples were carefully removed and washed with deionized water to clean the surface of the dye, placed on slides, and sealed with coverslips. They were observed within 1 h *via* CLSM (Nikon Eclipse Ti, Japan) using a complementary metal-oxide-semiconductor image sensor with an image resolution of 1024×1024. The light source is a laser with an excitation wavelength of 488 nm and an emission wavelength of 500—530 nm. After setting the parameters, use the 4× objective lens was used to find the sample under the bright field and adjust the position and lens focal length to bring the image to the middle of the field of view. Then, the 20× objective lens was applied, and the appropriate brightness of the field of view was adjusted. After this, the 60× oil lens was used, and mirror oil was dripped onto the surface of the objective lens. Next, the brightness of the field of view and the lens focal length were adjusted to clarify the image; finally, the bright field was closed, the laser was turned on, and the appropriate laser intensity was chosen to take the fluorescent image of the root hair cells.

#### 2.3.2 Dataset production

A dataset was produced using test samples from the confocal laser scanning microscope. The 8294 images that were obtained from sampling were used to create a dataset of which the ratio of the training set to the test set was 100:45, which meets the requirements of deep learning for the training and test sets without interfering with either. We selected 200 of these images as the set for training the model in each batch. Then, 90 were used as the test set to evaluate the model reconstruction effect.

#### 2.3.3 Model training and testing

Both models were implemented using the PyTorch framework. The training and testing processes were performed on the training and test sets, respectively, using Ubuntu on an Intel E5-2678 v3 64 G 1 TB + 256 G solid-state computer that had a dual NVIDIA 2080Ti (video memory 12 G).

The training learning rate of the model was set to 2×10^-4^, and the backpropagation algorithm adopted was Adam (Kingma and Jimmy, 2015). First, the training set images were input into the model in batches for training: 200 images were randomly selected for training in each batch, and 200 batches of training were carried out to obtain effective weights.

The reconstruction effect of the model was evaluated based on the test set.

#### 2.3.4 Evaluation of reconstruction quality

Combined with the dataset images and the reconstructed images during training and testing, the super-resolution reconstruction process and the post-reconstruction image quality of the SRGAN and SRResNet models were evaluated by obtaining the values for PSNR, SSIM, and MOS and the time required for the reconstruction process. The MOS was determined as the average result provided by the 26 selected professionals after scoring the reconstructed images obtained in different ways.

## 3 Results and discussion

To obtain and analysis a high-resolution reconstructed image, 4× image reconstruction (4×, 4 times the width and height) as single reconstruction and 16× image reconstruction (16×, 16 times the width and height) as secondary reconstruction were used here.

Using a learning rate of 2×10^-4^ and the Adam back-propagation algorithm, 200 images were randomly selected from the training set in each batch, and the training images were input into the SRGAN and SRResNet models implemented using PyTorch. After 200 training batches were completed, effective weight parameters were obtained when the loss function was 0.0164.

### 3.1 Super-resolution reconstruction of laser confocal images

**Figure 3:**
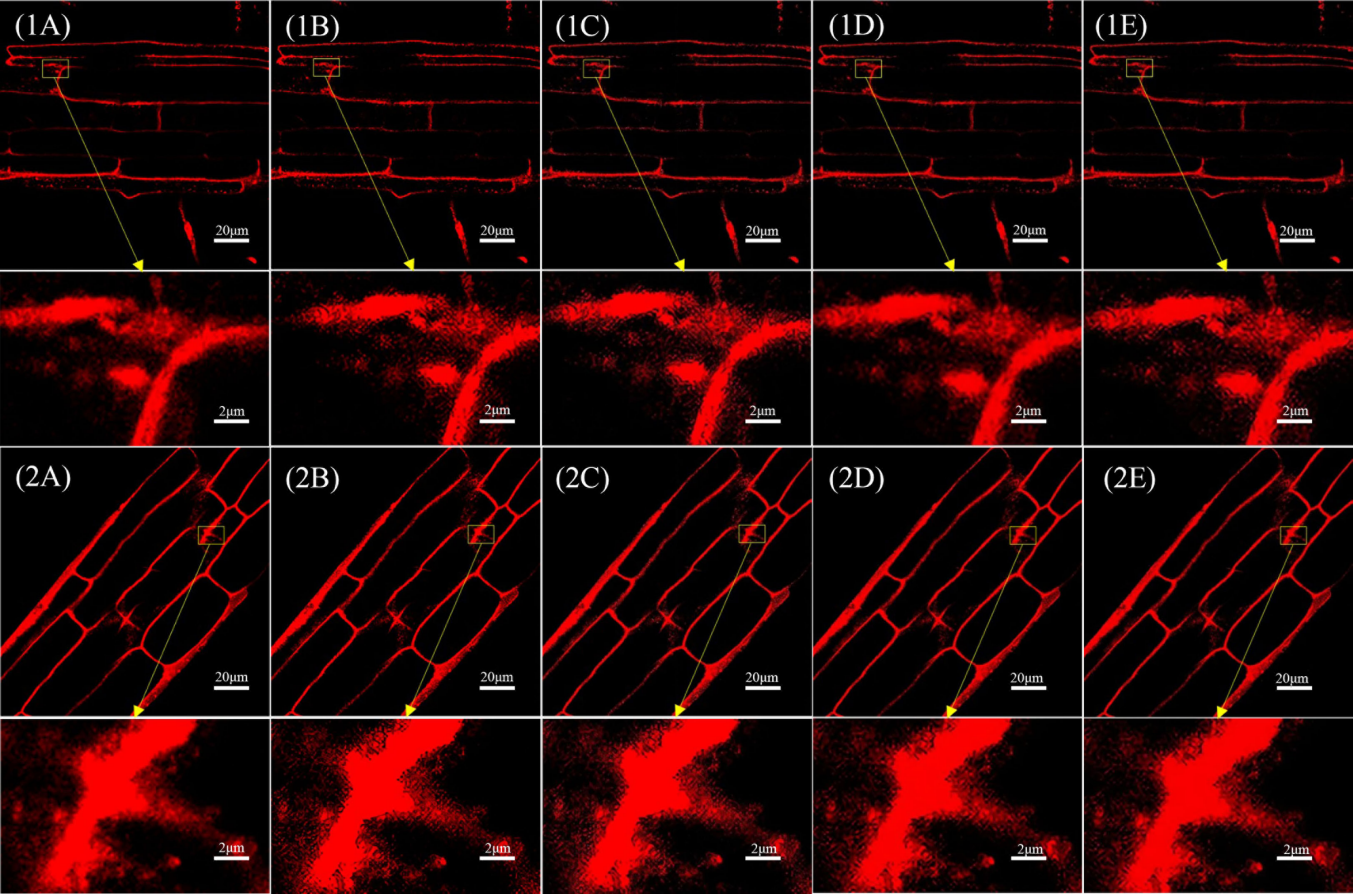
Comparison of single and secondary reconstruction images of different models in different images. **(1A)** Original image 1(pixel: 1024×1024) and local magnification map(pixel: 102×66). **(1B)** SRGAN single-reconstruction image(pixel: 4096×4096) and local magnification map(pixel: 410×264). **(1C)** SRGAN secondary-reconstruction image(pixel: 16384×16384) and local magnification map(pixel: 1638×1056). **(1D)** SRResNet single-reconstruction image(pixel: 4096×4096) and local magnification map(pixel: 410×264). **(1E)** SRResNet secondary-reconstruction image(pixel: 16384×16384) and local magnification map(pixel: 1638×1056). **(2A)** Original image 2(pixel: 1024×1024) and local magnification map(pixel: 102×66). **(2B)** SRGAN single-reconstruction image(pixel: 4096×4096) and local magnification map(pixel: 410×264). **(2C)** SRGAN secondary-reconstruction image(pixel: 16384×16384) and local magnification map(pixel: 1638×1056). **(2D)** SRResNet single-reconstruction image(pixel: 4096×4096) and local magnification map(pixel: 410×264). **(2E)** SRResNet secondary-reconstruction image(pixel: 16384×16384) and local magnification map(pixel: 1638×1056).

In all, 90 laser confocal images of the test set were input into the trained SRGAN and SRResNet models. **Figure 3** shows the results and comparison of the original image, single reconstruction image, secondary reconstruction image, and local magnification 10× maps corresponding to the two kinds of images.

**Table 1** compares the quality evaluation indicators (PSNR, SSIM, MOS) and time required for those reconstruction of different models (see **Appendix for MOS evaluation**) for the images in **Figure 3**. The MOS values of the images reconstructed by SRGAN and SRResNet were larger than those of the original image, with a maximum increase of 15.6%. The PSNR values were at a minimum of 37.268 and a maximum of 47.690, and the image quality was greatly improved. The PSNR and SSIM of the images following the secondary reconstruction of the same model were higher than those following the single reconstruction, with maximum increases of 21.7% and 2.8% for PSNR and SSIM, respectively, following secondary reconstruction. The MOS values of the secondary reconstruction images of different models were lower than those of the single reconstruction, and the time cost of the secondary reconstruction process was far higher than that of the single reconstruction. The time-consuming ratio of secondary and single reconstructions was greater than 130:1, and the maximums for SRGAN and SRResNet were 139.1 and 130.8, respectively. These results indicate that the reconstructions significantly improved image quality, signal-to-noise ratio, and image contrast; however, multiple reconstructions could reduce perceived quality to a certain extent, and result in a very large time cost.

**Table 1:** Comparison of the quality evaluation indicators and time required for different reconstruction.

Image	PSNR*/*dB	SSIM	MOS	Time (s)
1A (original image 1)			3.385	
1B (SRGAN 4×)	37.465	0.943	3.885	27.414
1C (SRGAN 16×)	38.982	0.952	3.769	3813.655
1D (SRResNet 4×)	39.293	0.967	3.615	29.886
1E (SRResNet 16×)	47.587	0.994	3.538	3852.368
2A (original image 2)			3.462	
2B (SRGAN 4×)	37.268	0.935	3.846	26.965
2C (SRGAN 16×)	39.673	0.960	3.731	3592.584
2D (SRResNet 4×)	39.192	0.967	3.692	27.720
2E (SRResNet 16×)	47.690	0.994	3.577	3625.298

For the same reconstruction times, the PSNR and SSIM values were higher in SRResNet than in SRGAN, the MOS values were lower in SRResNet than in SRAGN, and the time costs were similar between the two. This indicates that SRResNet had superior performance in improving the signal-to-noise ratio and image contrast, but had inferior perceived quality to that of SRGAN.

### 3.2 Reconstruction and recognition of endocytic vesicles in *Solanum nigrum* root cells

**Figure 4:**
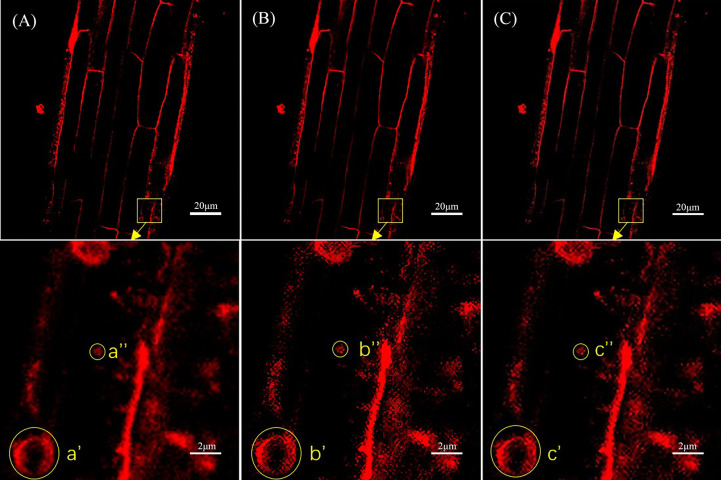
Comparison of the three types of images of *Solanum nigrum* root cells containing endocytic vesicles. **(A)** Original image(pixel: 1024×1024) and local magnification map(pixel: 102×102). **(B)** SRGAN single-reconstruction image(pixel: 4096×4096) and local magnification map(pixel: 410×410). **(C)** SRResNet single-reconstruction image(pixel: 4096×4096) and local magnification map(pixel: 410×410).

**Figure 4** shows a comparison of the original laser confocal image, SRGAN reconstruction image, and SRResNet reconstruction image of *Solanum nigrum* root cells containing endocytic vesicles and the 10× local magnification maps of endocytic vesicles. The image qualities of the SRGAN and SRResNet reconstructions were better than that of the original image. Further, the boundary clarities of endocytic vesicles were significantly better than those in the original image.

**Figure 5:**
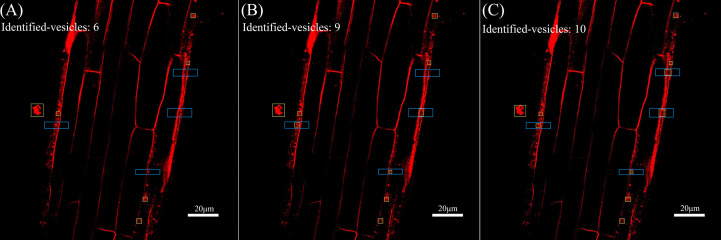
Comparison of recognition of endocytic vesicles in laser confocal images of *Solanum nigrum* root cells. **(A)** Recognition of endocytic vesicles in original image(pixel: 1024×1024). **(B)** Recognition of endocytic vesicles in SRGAN single-reconstruction image(pixel: 4096×4096). **(C)** Recognition of endocytic vesicles in SRResNet single-reconstruction image(pixel: 4096×4096).

Next, the reconstructed images were automatically identified using YOLOv5 for endocytic vesicles using the same model parameters and weights. **Figure 5** shows the quantitative recognition effect corresponding to the original image, the SRGAN single reconstruction image, and the SRResNet single reconstruction image. Endocytic vesicles are marked with yellow boxes and the regions where the extra vesicles were identified by the two models are marked with blue boxes.

Six, nine, and ten endocytic vesicles were identified in the three images, respectively. These results indicate that the reconstruction effectively improved the quantitative recognition effect, confirming the improvement of image quality. The image details were richer, which would aid the mining of the deep-level information contained in the image. The recognition effect of the SRResNet-reconstructed image was better than that of the SRGAN-reconstructed image.

Finally, the mean fluorescence intensity of each image shown in **Figure 5** was analyzed using ImageJ. The intensities were 175.654, 200.981, and 189.387 for the original image, the SRGAN reconstruction image, and the SRResNet reconstruction image, respectively, enhanced by 14.4% and 7.8% by the two models,respectively.

## 4 Conclusion

Super-resolution reconstruction technology based on deep learning can be used in the study of laser confocal images. Single reconstruction (4×) and secondary reconstruction (16×) using the SRGAN and SRResNet models significantly improved the evaluation indicators PSNR, SSIM, and MOS of the image as well as the resolution. The maximum PSNR was 47.690, indicating that the quality of reconstructed image was significantly improved. The PSNR and SSIM of the secondary reconstruction image were obviously better than those of the single reconstruction, with maximum increases of 21.7% and 2.8%, respectively. Additionally, the objective evaluation of the image quality was good, but the MOS was generally lower than that of the single reconstruction, and the perceptual quality was weakened. The reconstruction effect using SRResNet was better than that obtained using SRGAN. The images reconstructed by SRGAN and SRResNet also clarified the boundaries of endocytic vesicles in *Solanum nigrum* root cells. They also revealed more vesicles (9 and 10, respectively, versus only 6 in the original image). Finally, the mean fluorescence intensity was enhanced by 14.4% and 7.8%, which indicates the feasibility of deep mining of the embedded information in laser confocal images.

## Data availability statement

The raw data supporting the conclusions of this article will be made available by the authors, without undue reservation.

## Author contributions

FH and FW conceived and designed the research, and guided the preparation and writing of the manuscript. WL searched for relevant literature, carried out super-resolution reconstruction of images based on laser confocal images, and wrote the manuscript. DH and YL sampled and collected those laser confocal images of hyperaccumulator *Solanum nigrum* endocytosis vesicles. All authors contributed to the article and approved the submitted version.
