# Supplementary material for: Super-resolution reconstruction, recognition, and evaluation of laser confocal images of hyperaccumulator Solanum nigrum endocytosis vesicles based on deep learning: Comparative study of SRGAN and SRResNet
Source: Front Plant Sci. 2023 Mar 21;14:1146485. doi: 10.3389/fpls.2023.1146485 (PMC10070864; doi:10.3389/fpls.2023.1146485)
Supplement: Supplementary file 1 [file Table_1.docx]

Supplementary Material

**Super-resolution reconstruction, recognition, and evaluation of laser confocal images of hyperaccumulator Solanum nigrum endocytosis vesicles based on deep learning: comparative study of SRGAN and SRResNet**

**Wenhao Li ^1†^, Ding He ^2^, Yongqiang Liu ^2^, Fenghe Wang ^2*†^, Fengliang Huang ^1*†^**

^1^ School of Electrical and Automation Engineering, Nanjing Normal University, Nanjing, China

^2^ Jiangsu Provincial Key Laboratory of Materials Cycling and Pollution Control, School of Environment, Nanjing Normal University, Nanjing, China

***Correspondence:**Fengliang Huang: huangfengliang@njnu.edu.cn, Fenghe Wang: wangfenghe@njnu.edu.cn

† These authors have contributed equally to this work.

# Supplementary Data

**Appendix: Evaluation score of each expert in MOS evaluation**

| **Image**  **Number**  **Serial Number** | **1A** | **1B** | **1C** | **1D** | **1E** | **2A** | **2B** | **2C** | **2D** | **2E** |
| --- | --- | --- | --- | --- | --- | --- | --- | --- | --- | --- |
| 1 | 3 | 4 | 4 | 3 | 3 | 3 | 4 | 4 | 4 | 3 |
| 2 | 3 | 3 | 4 | 3 | 3 | 3 | 3 | 3 | 3 | 3 |
| 3 | 4 | 4 | 4 | 4 | 4 | 4 | 5 | 4 | 4 | 4 |
| 4 | 4 | 3 | 4 | 4 | 4 | 3 | 3 | 3 | 3 | 3 |
| 5 | 4 | 4 | 4 | 3 | 3 | 4 | 4 | 4 | 4 | 4 |
| 6 | 3 | 4 | 3 | 4 | 4 | 3 | 4 | 4 | 4 | 4 |
| 7 | 3 | 3 | 3 | 3 | 3 | 4 | 5 | 4 | 4 | 4 |
| 8 | 4 | 4 | 4 | 4 | 4 | 3 | 3 | 4 | 4 | 4 |
| 9 | 3 | 4 | 3 | 3 | 3 | 4 | 4 | 3 | 3 | 3 |
| 10 | 3 | 4 | 4 | 4 | 4 | 4 | 4 | 4 | 4 | 4 |
| 11 | 4 | 5 | 4 | 4 | 4 | 4 | 4 | 4 | 4 | 4 |
| 12 | 3 | 3 | 3 | 3 | 3 | 3 | 3 | 4 | 4 | 4 |
| 13 | 4 | 4 | 4 | 4 | 3 | 2 | 4 | 4 | 4 | 4 |
| 14 | 4 | 4 | 4 | 3 | 3 | 3 | 4 | 3 | 3 | 3 |
| 15 | 3 | 4 | 4 | 4 | 4 | 4 | 4 | 4 | 4 | 4 |
| 16 | 4 | 5 | 4 | 4 | 4 | 4 | 5 | 5 | 4 | 4 |
| 17 | 3 | 4 | 4 | 4 | 4 | 3 | 3 | 4 | 3 | 3 |
| 18 | 3 | 4 | 4 | 4 | 3 | 4 | 4 | 3 | 3 | 3 |
| 19 | 4 | 5 | 4 | 4 | 4 | 3 | 4 | 4 | 4 | 3 |
| 20 | 3 | 4 | 4 | 4 | 4 | 3 | 4 | 4 | 4 | 4 |
| 21 | 4 | 4 | 4 | 4 | 4 | 4 | 4 | 4 | 3 | 3 |
| 22 | 2 | 3 | 3 | 3 | 3 | 4 | 4 | 4 | 4 | 4 |
| 23 | 3 | 4 | 4 | 4 | 4 | 3 | 3 | 3 | 4 | 3 |
| 24 | 4 | 4 | 4 | 3 | 3 | 4 | 3 | 3 | 4 | 4 |
| 25 | 3 | 3 | 3 | 3 | 3 | 3 | 4 | 4 | 4 | 4 |
| 26 | 3 | 4 | 4 | 4 | 4 | 4 | 4 | 3 | 3 | 3 |
| Average | 3.385 | 3.885 | 3.769 | 3.615 | 3.538 | 3.462 | 3.846 | 3.731 | 3.692 | 3.577 |
